# Biological, genetic, neurological and environmental influences on homosexuality—a narrative review

**DOI:** 10.3389/fnbeh.2025.1574713

**Published:** 2025-09-23

**Authors:** Michel Alagha, Freddy Antoun, Christine Bacha, Tiara El Nabbout, Noura B. El Khoury

**Affiliations:** ^1^Faculty of Medicine and Medical Sciences, University of Balamand, El Koura, Lebanon; ^2^Department of Psychology, Faculty of Arts and Sciences, University of Balamand, El Koura, Lebanon

**Keywords:** homosexuality, LGBT, biological factors, environmental factors, neurologic factors, genetic factors, sexual orientation

## Abstract

Homosexuality is an intricate and multifactorial phenomenon affected by the interaction of biological, genetic, neurological and environmental factors. This paper examines the interplay of homosexuality determinants. Biological determinants such as the role of androgen levels, the fraternal birth order effect and maternal immune response contribute to shaping sexual orientation. Additionally, genetic influences are also assessed. These include the potential role of X chromosome, the possible link of fragile X mental retardation neighbor gene (FMR1) to sexual orientation, the function of genetic variants such as COMT an MTHFR, as well as connection with chromosomes 7, 8, 13 and 14. Furthermore, neurologic factors such as the role of the hypothalamus are assessed to highlight their contribution to sexual preference and attraction mediation. Lastly, childhood gender nonconformity and early exposure to traumatic events are among the environmental influences that contribute to the development of homosexuality. By incorporating various perspectives, this paper seeks to present a thorough overview of the multiple factors influencing sexual orientation, while emphasizing the importance of ongoing interdisciplinary research in this area.

## 1 Introduction

Human Sexual behavior is complex practice that depends on the processing of sexual stimuli that allow individuals to enter the sexual cycle. From an evolutionary perspective, this behavior is fundamental, supporting reproductive interactions that are vital for biological adaptation and species survival (Calabrò et al., 2019). The regulation of sexual behavior involves both subcortical structures, including the hypothalamus, brainstem, and spinal cord, as well as various cortical brain areas that orchestrate together to finely tune this primitive yet complex behavior. Central to this regulation are the dopaminergic and serotonergic systems, which play significant roles in different aspects of sexual response, although adrenergic, cholinergic, and other neuropeptide transmitter systems may also contribute (Calabrò et al., 2019). In humans, sexual behavior should be viewed as a pleasure-seeking impulse that can be appropriately controlled under the influence of cultural factors such as morality and ethics (Calabrò et al., 2019).

Sexual orientation refers to the enduring sexual attraction towards individuals of the opposite sex (heterosexuality), the same sex (homosexuality), both sexes (bisexuality), or a lack of interest in either sex (asexuality) following maturity (Rosario and Schrimshaw, 2014). It is distinct from related concepts like sexual partner preference, gender identity, and sexual behavior (Rosario et al., 2006). Sexual partner preference, which is highly sexually dimorphic, describes an animal’s sexual attraction to a partner of either the same or opposite sex when given a choice. Gender identity is the self-perception of being male, female, both, or neither, while sexual behavior refers to the actual sexual interactions an individual engages in (Bailey et al., 2016; Bailey and Zuk, 2009). Gender identity is an individual’s deeply held internal sense of being female, male, neither, both, or fluid, which may not be outwardly visible and may not align with the sex assigned at birth. It can evolve and change over a person’s life (Ervin et al., 2023). While sexual orientation and gender identity are specific to humans, sexual partner preference and behavior are observed in both humans and other animals.

Sexual orientation, which refers to predisposed sexual behavior, constitutes a fundamental aspect of individual sexual identity (Rahman, 2005a, 2005b). It encompasses enduring patterns of emotional, romantic, and/or sexual attractions to either men, women, or both genders (Roselli, 2018). Research interest in understanding the etiology of male homosexuality has increased significantly over the past decade (Qin et al., 2018).

Homosexuality, defined as the romantic and sexual attraction to individuals of the same gender, has undergone linguistic evolution since its initial introduction in 1868 within the field of sexology (Herek and Garnets, 2007). The term “lesbian,” tracing its roots back to the Greek poet Sappho from the island of Lesbos, similarly underwent linguistic shifts during the 19th century (Duden, 1989). In contrast, the term “gay,” derived from Old French (“gai”) and Germanic languages, has a longer history dating back to the 13th century (Collins, 1995).

Twin studies and segregation analyses have revealed that homosexuality is influenced by a complex interplay of biological, genetic, neurological and environmental factors (Hu et al., 2021; Kendler et al., 2000; Bailey and Pillard, 1991). Genetic examinations rooted in behavioral sciences have provided substantial evidence for the heritability of sexual orientation, estimating genetic influence at approximately 32% (Sanders et al., 2021; Bailey et al., 2016). Multiple biological factors contribute to the occurrence of homosexuality, including prenatal sex hormones, particularly testosterone, which shapes behavioral sex differences across various species (Roselli, 2018). Neural circuitry present during early fetal development is also believed to play a role in the continuum of sexual orientations (Rahman, 2005a, 2005b). Fraternal birth order has been linked to male sexual orientation, possibly due to maternal immunization processes affecting sexual differentiation in fetal life and differing social environments experienced by younger brothers (Rahman, 2005a, 2005b). Additionally, progressive immunization of some mothers to male-specific antigens with each successive male fetus may impact brain sexual differentiation, potentially influencing sexual orientation (Gavrilets and Rice, 2006). Social experiences and sociocultural factors also contribute to the development of homosexuality (Hu et al., 2021; Kendler et al., 2000; Bailey and Pillard, 1991). Psychoanalytic theories further underscore the role of family experiences in shaping same-sex behavior (Qin et al., 2018; Friedman, 1988). Despite all these studies, mechanisms underlying homosexuality remain poorly understood.

In this review, we aim to develop the biological, genetic, neurological and environmental factors that contribute to the development of homosexuality. To our knowledge, this is the first review in the middle east region that discusses this topic.

## 2 Materials and methods

This review was developed using a narrative approach to synthesize existing literature on the biological, genetic, neurological, and environmental factors influencing homosexuality. A thorough search was performed across three major databases: PubMed, Google Scholar, and ScienceDirect. The search strategy utilized a combination of keywords and MeSH terms, including: homosexuality, sexual orientation, LGBT, biological influences, genetic markers, neurological mechanisms, environmental contributors, prenatal hormones, COMT, MTHFR, fraternal birth order, and gender nonconformity. A total of 63 articles were reviewed in this narrative synthesis. Of these, 17 addressed biological factors, 17 examined genetic influences, 15 explored neurological mechanisms, and 14 investigated environmental determinants. The search focused on studies published in English from 1980 to 2024, encompassing both human and relevant animal research. Inclusion criteria were based on the relevance of the study to at least one of the four domains examined in this review. Priority was given to peer-reviewed empirical studies, genome-wide association studies (GWAS), neuroimaging analyses, twin studies, and systematic reviews. Additional articles were identified by manually reviewing the reference lists of key sources. Although no formal quality assessment tool was applied, studies were selected based on methodological soundness and their contribution to understanding the multifactorial nature of sexual orientation.

## 3 Influencing factors on homosexuality

### 3.1 Biological factors

#### 3.1.1 The effect of androgen levels

##### 3.1.1.1 In females

The theory of prenatal androgens suggests that gonadal androgens play a significant role in determining the human sexual orientation. The conducted research explains the different observations in brain structure and behavior between vertebrates (Morris et al., 2004). This theory is supported by preclinical (animals) and clinical studies (Humans).

###### 3.1.1.1.1 Pre-clinical studies

The theory of prenatal androgens received initial backing from experiments involving the manipulation of prenatal sex hormones in animals. In mammals, hormones from the testes, such as androgens or estrogens derived from androgens, influence male differentiation during prenatal or early postnatal stages. These hormones can enhance male-typical behaviors, like mounting, or reduce female-typical behaviors, like lordosis, or both. The significance of masculinization versus defeminization in male development varies by species and depends on whether male or female behaviors are more distinctly different (Baum, 1979). In the absence of testicular hormones, female differentiation occurs, suggesting that ovaries are not essential for developing female behaviors. Experimental evidence shows that females exposed to testosterone or estradiol early in development exhibit male-like behaviors such as increased mounting after testosterone priming and decreased receptivity after estradiol or a combination of estradiol and progesterone priming. Conversely, males that are castrated early develop female-like characteristics, showing reduced mounting and heightened receptivity after appropriate hormone treatments. These effects from early hormonal interventions are permanent and typically occur during a critical developmental period, leading to their classification as “organisational” effects, in contrast to the temporary “activational” effects of the hormones given in adults. Research on the organisational effects of sex hormones on mating behavior has shown that male-typical and female-typical behaviors are distinct dimensions. It is possible for masculinization to occur without defeminization and vice versa (Feder, 1981).

###### 3.1.1.1.2 Clinical studies

In one study, Manning (2002) concluded that elevated levels of prenatal steroid hormones may play a role in the sexual orientation of “butch lesbians.” A “butch lesbian” is a woman who identifies as a lesbian and expresses her gender in a masculine way. This often means she adopts traditionally masculine characteristics in how she looks and behaves. Research shows this is a unique form of female masculinity found within lesbian communities (Rosario et al., 2011). Retrospective indicators of prenatal androgen exposure, such as digit ratios suggest that, on average, lesbians experienced higher levels of prenatal androgens compared to straight women (Breedlove, 2017). Specifically, lesbians that identify as “butch” have more masculine digit ratios compared to those identifying as “femme” suggesting an even higher exposure to prenatal hormones (Brown et al., 2002a). In simple terms, a “femme” is a lesbian woman who expresses her gender in a way that’s traditionally seen as feminine. This often means she enjoys presenting with things like dresses, makeup, and longer hairstyles, which are usually associated with femininity (Maltry and Tucker, 2002). Although digit ratios and long-bone development are frequently used as indicators of prenatal androgen exposure, these measures are indirect and may be influenced by a range of postnatal or ethnic factors. Furthermore, the available evidence is largely correlational and retrospective in nature, which limits the strength of any causal inferences drawn from these associations.

The findings are backed by information about the frequency of play behaviors typically associated with males, decreased contentment with being assigned a female sex at birth, diminished interest in heterosexual activities during childhood and adolescence, and eventual engagement in lesbian relationships among women diagnosed with congenital adrenal hyperplasia (e.g., Money et al., 1984; Hines et al., 2004). Singh et al. (1999) found that self-identified “butch lesbians” recalled exhibiting more childhood behaviors not conforming to traditional gender roles such as playing with boy’s toys instead of girl’s toys. This possibly suggests that they might not see themselves as females but rather as males. They also expressed less desire to have children and had higher waist-to-hip ratios and testosterone levels compared to self-identified “femmes” (Pearcey et al., 1996). Research has identified a link between elevated androgen levels and increased waist-to-hip ratio (WHR) in various groups, including postmenopausal women, women with androgen excess, obese adolescent girls, female-to-male transgender individuals, and women of different sexual orientations. Evidence from female-to-male transgender individuals indicates that testosterone treatment can lead to an increase in abdominal visceral fat without hip fat deposition, resulting in a higher WHR proving that a high WHR is a male characteristic related to a high androgen state (Van Anders and Hampson, 2005). However, the cellular mechanisms by which androgens influence fat distribution and elevate WHR remain poorly understood (Van Anders and Hampson, 2005).

“Proxy markers” for prenatal hormonal exposure have been researched. These are non-invasively explored in the endocrinologically normal population and they consist of somatic features that are understood to be shaped by prenatal exposure to sex hormones. Morris et al. (2004) pointed out that there are three indicators that are believed to indicate the presence of fetal androgens in lesbians that have been more exposed to them in utero than straight women. The indicators are: (1) click-evoked otoacoustic emissions (McFadden and Pasanen, 1998); (2) eye blink reflexes (Rahman et al., 2003a, 2003b, 2003c); and (3) finger length ratios. Therefore, demonstrating differences in these characteristics among adult heterosexuals and homosexuals could offer insight into the early development of sexual preferences influenced by prenatal hormones.

Click-evoked otoacoustic emissions, known as CEOAEs, are echo-like waveforms emitted by cochlea of individuals with normal hearing in response to a brief stimulus. Research indicates that CEOAEs tend to be more pronounced in females compared to males. The CEOAEs of homosexual and bisexual females were intermediate between those of heterosexual females and males. This suggests that the auditory systems and relevant brain structures related to sexual orientation in homosexual and bisexual females may have undergone partial masculinization due to prenatal exposure to elevated androgen levels. Interestingly, no distinction in CEOAEs was noted between homosexual and heterosexual males (McFadden and Pasanen, 1998).

Prepulse inhibition (PPI) denotes a decrease in the startle response triggered by a strong sensory stimulus when preceded by a weaker stimulus, known as the prepulse. It reflects an innate mechanism for regulating sensorimotor functions and exhibits a noticeable gender contrast, with women typically demonstrating lower PPI compared to men. The eyeblink startle responses to auditory stimuli in 59 healthy individuals, encompassing both heterosexual and homosexual men and women revealed that homosexual women displayed a notably more masculinized pattern of PPI compared to heterosexual women, while no notable difference in PPI was observed between homosexual and heterosexual men. These findings provide evidence for within-gender variations in fundamental sensorimotor gating mechanisms and suggest involvement of established neural pathways associated with PPI in human sexual orientation (Rahman et al., 2003a, 2003b, 2003c).

One commonly used “proxy marker” of prenatal hormonal exposure is the second to fourth finger length ratio, known as the 2D:4D ratio (Manning et al., 1998). The presence of reduced ratios in individuals exposed to excess androgens, as seen in conditions like congenital adrenal hyperplasia, strongly suggests that prenatal androgens play a significant role in influencing the 2D:4D ratio (Brown et al., 2002b; Buck et al., 2003). 2D:4D is also linked to variation in the androgen receptor gene (Manning et al., 2003) and the ratio of testosterone to estrogen taken from amniotic fluid during gestation is negatively associated with 2D:4D at 2 years of age (Lutchmaya et al., 2004). Although ultimately correlational, these data strongly indicate that an overabundance of androgens can influence the relative lengths of the second and fourth finger digits. Four separate studies have demonstrated that homosexual women tend to have notably more masculine (lower) 2D:4D ratios compared to heterosexual women, although it seems that this effect is hand-specific (Rahman and Wilson, 2003a, 2003b; Williams et al., 2000; Rahman, 2005a, 2005b; McFadden and Schubel, 2002).

In 1981, Perkins reported that exclusively homosexual women categorized as ‘dominant’ were notably taller, had broader shoulders, and narrower hips compared to those categorized as ‘passive’. Inconsistent findings have emerged regarding self-reported height and weight differences related to sexual orientation (Bogaert and Friesen, 2002; Bogaert et al., 2002). However, it remains uncertain whether these variations are primarily due to prenatal sex steroids or a combination of postnatal factors. One recent study has found that homosexual women have more long-bone growth in the arms, legs and hands compared to heterosexual women (Martin and Nguyen, 2004). During childhood, certain bone characteristics display sexual differences in homosexual individuals, but this distinction diminishes after puberty. These findings imply that homosexual women exhibit partial masculinization in specific anthropometric measures prior to the increase in sex steroid levels during puberty (Martin and Nguyen, 2004).

Similarly, Over-exposure to prenatal androgens leads to homosexuality in women (Ellis and Ames, 1987). Moreover, this classic model of the origins of sexual orientation is supported from the observation of differing sexual orientations in cases of human intersex individuals or those with endocrine disorders like congenital adrenal hyperplasia, which align with the idea of hormonal exposure during prenatal development (Bailey, 2003; Morris et al., 2004). Congenital adrenal hyperplasia (CAH) is a genetic disorder affecting the steroid-producing enzymes in the adrenal cortex, resulting with excess androgens. Depending on the presence of androgens, the fetal brain can develop in a typically male direction suggesting that aspects of sexual development, including sexual orientation, may be influenced by brain structure programming during prenatal development (Daae et al., 2020). Systematic reviews investigated sexual orientation in females with CAH. A review included a sample of 927 assigned females at birth. Twelve studies with control groups indicated that the prevalence of non-heterosexual orientation was greater among assigned females with CAH compared to the control group (Money et al., 1984; Dittmann et al., 1992; Kuhnle et al., 1993; Zucker et al., 1996; Hines et al., 2004; Johannsen et al., 2006; Meyer-Bahlburg et al., 2008; Frisen et al., 2009; Fagerholm et al., 2011; Lesma et al., 2014; Binet et al., 2016; Khorashad et al., 2017).

All research shows that homosexual women are exposed to more prenatal androgens compared to heterosexual women; it was also observed that elevated levels of prenatal estrogen also have been linked to the development of lesbian orientation (Meyer-Bahlburg et al., 1995; Collaer and Hines, 1995).

##### 3.1.1.2 In males

There is increasing evidence suggesting that certain genetic factors contribute to the sexual orientation of some homosexual men. Under-exposure to prenatal androgens leads to homosexuality in men, however, bisexual males are thought to have been exposed to increased prenatal testosterone levels. On average, bisexual men are reported to have a younger age of first sexual experience, tend to be taller than heterosexual men (Bogaert and Friesen, 2002), have a higher sex drive than other men on the average (Comings, 1994) and are more likely to participate in sexual activities with higher levels of risk than control homosexuals or heterosexuals (Doll and Beeker, 1996) with the latter being associated with higher testosterone levels (Zuckerman, 1994). As mentioned above with lesbians, finger-length ratio and higher uterine testosterone levels exposure are seen in bisexual men as well as higher testosterone levels in infancy, childhood, adolescence, and adulthood. Research also found that homosexual men reach puberty earlier than heterosexual men (Bogaert et al., 2002); during a period when males are still predominantly interacting with each other, making it more probable for them to associate their sexual awakening with males rather than females (Storms, 1981; Wyre, 1990). One commonly used “proxy marker” of prenatal hormonal exposure is 2D:4D ratio (Manning et al., 1998). According to this ratio, bisexual males have lower ratios than females (Rahman and Wilson, 2003a, 2003b; Williams et al., 2000; Rahman, 2005a, 2005b; McFadden and Schubel, 2002). One study has found that homosexual men have less long-bone growth in the arms, legs and hands compared to heterosexual men (Martin and Nguyen, 2004). During childhood, certain bone characteristics display sexual differences in homosexual individuals, but this distinction diminishes after puberty. These findings imply that homosexual men exhibit partial feminization prior to the increase in sex steroid levels during puberty (Martin and Nguyen, 2004). Research shows that homosexual men exhibit mosaic traits ranging from sex-typical to sex-atypical and other sex-exaggerated factors. Differences in the timing and amount of androgen exposure, such as below-average or above-average levels, could potentially lead to this variation in heterosexual and homosexual males. For example, Geschwind and Galaburda (1985) proposed that homosexual men might experience elevated androgen levels during early development. On the other hand, homosexual men have a 34% chance of being non-right-handed compared to their heterosexual counterparts while homosexual women have a 91% chance (Lalumiere et al., 2000). Thus, a correlation between homosexuality and being left-handed has emerged. This explanation could be found in developmental instability (DI), which refers to how vulnerable an organism is to environmental and genetic stresses during its development. According to this view, same-sex orientation may result from generalized developmental challenges that push sexual preferences away from the typical pattern of opposite-sex attraction (Lalumiere et al., 2000).

#### 3.1.2 The fraternal birth order

Epidemiological research has consistently indicated that having older brothers raises the likelihood of homosexuality in younger males (Blanchard, 1997; Blanchard and Ellis, 2001) but not in females. This is known as the “fraternal birth order (FBO) effect” (Blanchard and Ellis, 2001). The FBO effect has been observed in various homosexual individuals, including both those from typical community volunteers and those from atypical groups, who have diverse characteristics and preferences in their desired partners (Blanchard and Bogaert, 1996a, 1996b; Blanchard et al., 1998; Ellis and Blanchard, 2001; Robinson and Manning, 2000; Blanchard and Bogaert, 1997; Purcell et al., 2000; Williams et al., 2000). In males, each extra older brother raises the likelihood of homosexuality by around 33% but there no link was observed in older sisters. Nevertheless, this FBO effect explains the sexual orientation of only some gay men (Bogaert and Skorska, 2011; Blanchard and Bogaert, 1996a). By contrast, there is no variation neither in birth order nor in sibling composition between homosexual and heterosexual women (Bogaert, 1997). According to Manning (2002), approximately one in seven homosexual men associate their sexual orientation to the FBO effect (Cantor et al., 2002). The fraternal birth order effect appears to explain only a subset of male homosexuality and has not been observed in females. Additionally, reliance on self-reported familial data introduces potential recall bias. Effect sizes reported in the literature are often modest, and social or environmental variables linked to birth order are frequently not adequately controlled.

##### 3.1.2.1 Antibodies against a Y-linked factor

The primary theory proposing an explanation for the linkage of antibodies against the Y-linked factor suggests that certain mothers produce antibodies against a Y-linked factor which is crucial for the development of the male brain. This response appears to intensify with each subsequent male pregnancy, consequently resulting in changes in the brain structures that influence the sexual orientation of later-born boys. In support of this theory, Bogaert et al. demonstrated that mothers of homosexual sons, particularly those with older brothers, have higher antibody titers to neuroligin 4 (NLGN4Y), which is an extracellular protein involved in synaptic functioning and influencing fetal brain development.

Research has shown that gay men who have older brothers tend to have lower birth weights in comparison to straight men with older brothers (Blanchard and Ellis, 2001; Blanchard et al., 2002). Researchers have suggested that the repeated exposure to male-specific antigens during successive pregnancies may lead to a gradual immune response in some mothers as the maternal immune system identifies male-related antigens as foreign and starts generating antibodies against them (Blanchard, 2004). A potential set of antigens that can play a role here are the Y-linked minor histocompatibility antigens, particularly H-Y. As an illustration, male-specific antibodies might attach to and disable receptors responsible for masculinizing fetal neurons. This action can hinder the development of male-oriented sexual preferences. Neural tissue contains a significant number of H-Y antigens, and the H-Y antigen is exclusively found in male fetuses, so the maternal immune system retains a memory of past male pregnancies and can adjust its response accordingly (Blanchard, 2004; Blanchard and Bogaert, 1996a). Various other potential antigens besides H-Y are being considered, such as unique Y-linked protein groups like protocadherin and neuroligin, both of which have been identified in humans. These cell adhesion proteins are believed to affect communication between cells in the initial development of male-specific brain morphogenesis and could result in male-typical behavioral outcomes (Blanco et al., 2000). Also, the male human brain (including the hypothalamus) has a direct transcription of Y-linked sex determination genes SRY and ZFY (Mayer et al., 1998). While the maternal immune hypothesis offers an intriguing explanation, current findings are largely correlational and supported primarily by animal models. In humans, the evidence remains limited, and individual variability in maternal immune response adds complexity to establishing a consistent and generalizable mechanism.

##### 3.1.2.2 Pre-clinical rodent studies

These findings were supported by preclinical mice in rodents. For instance, a study conducted on mice revealed that maternal immunity to male-derived antigens can impact fetal weight (Gentile et al., 1992; Lu and Dawson, 1986). Likewise, male mice born to mothers who were immunized against H-Y before pregnancy display diminished male-typical consummatory sexual behavior when interacting with receptive females (Singh and Verma, 1987). It is unknown yet whether male-specific antibodies could potentially engage with sexual differentiation processes regulated by sex hormones or operate entirely autonomously from them. Although animal models provide valuable insights into the neurobiological underpinnings of sexual behavior, their applicability to humans is limited. Species differences in brain development and behavior, as well as the artificial nature of experimental manipulations, challenge the direct translation of findings to human sexual orientation.

##### 3.1.2.3 Sibling sex-play

Another possible explanation for the (FBO) effect is that interactions with older brothers during critical stages of sexual development might predispose individuals to a homosexual orientation. However, research conducted by Bogaert (2000) suggests that engaging in sibling sex-play is not a significant factor linking FBO to male sexual orientation (Bogaert, 2000). Instead, it is the frequency of having older brothers, rather than the specific sexual activities, that most accurately predicts the sexual attraction aspect of one’s sexual orientation (Bogaert, 2003). Additionally, the absence of a connection between same-sex play among pairs of gay brothers and later adult homosexual attraction has been noted (Dawood et al., 2000).

### 3.2 Genetic components

The analysis of the development of physiological and behavioral traits should always examine genetic factors. Many family and twin studies showed evidence of genetic influence on both male and female sexual orientation. Family studies showed a higher occurrence of homosexuality among relatives of homosexual people (Bailey and Pillard, 1995). The likelihood of genetic diversity differs in a significant manner between sex chromosomes and autosomes (Manning, 2002). However, it was found that the 50% similarity in sexual orientation among identical twins is not only due to genetic factors but also to other factors such as non-genetic elements and chance. Additionally, evidence suggests an increased likelihood of maternal transmission of male homosexuality, indicating a possible connection to the X chromosome (Camperio-Ciani et al., 2004; Hamer et al., 1993). However, other research has found no correlation with paternal transmission (Bailey et al., 1999). Females have a complex transmission pattern including both autosomal and sex-linked pathways (Pattatucci and Hamer, 1995). Moderate heritability estimates were shown in twin studies in community and population samples. Genes affecting sexual orientation can easily extend and be found in different forms under many conditions as indicated by the genetic models concerning homosexuality. The idea that alleles linked to homosexuality may provide a selective advantage when they are rare, while becoming disadvantageous when common, is vital for explaining how genetic diversity is maintained. This concept is backed by theories such as frequency-dependent selection and heterozygote advantage. Frequency-dependent selection suggests that the effectiveness of a trait is contingent on its frequency; when an allele is infrequent, it could facilitate enhanced social interactions, foster cooperation, or improve support among relatives, thereby boosting their reproductive success (Sasani and Gardner, 2020; Kirkpatrick and Barton, 1997). Furthermore, the heterozygote advantage implies that individuals with one copy of a certain allele may exhibit traits like enhanced creativity or social skills, which can indirectly benefit the reproductive success of their relatives (Hamer and Copeland, 1998; Rieger et al., 2008a, 2008b). As the frequency of the allele increases, its advantages might wane due to rising competition and evolving social dynamics, which plays a role in maintaining genetic diversity associated with homosexuality in populations (Hamer, 2007). Overall, these evolutionary dynamics ensure that such alleles remain present despite their potential reproductive drawbacks.

#### 3.2.1 The role of X chromosome

Polymorphism is generally less common on the X chromosome compared to autosomes. However, in the case of sexually antagonistic genes—those that exert different effects on males and females—the potential for polymorphism on the X chromosome becomes significantly higher, especially if these genes primarily influence masculine traits. Although this phenomenon is not fully understood, it suggests that if genes linked to homosexuality are located on the X chromosome, it would strongly support the theory of sexual antagonism over that of overdominance (Gavrilets and Rice, 2006). Sexual antagonism and overdominance are two evolutionary mechanisms that help maintain certain alleles in a population. Sexual antagonism occurs when an allele has different effects on males and females, benefiting one sex while disadvantaging the other. Overdominance, by contrast, refers to a heterozygote advantage, where individuals with one copy of an allele have greater fitness than those with two copies of either allele, preserving genetic diversity without regard to sex-specific effects. Since the X chromosome represents a small portion of the genome in most species, the presence of multiple X-linked gene loci associated with homosexuality would emphasize sexual antagonism as a key factor in maintaining genetic variation related to homosexuality.

In the study of the genetics of homosexuality, researchers have mapped male sexual orientation to the Xq28 chromosomal region using microsatellite markers. Hamer et al. (1993) identified genetic markers in this region, although later studies confined this effect to males (Hu et al., 1995). A more recent genome-wide scan, however, did not confirm the Xq28 linkage in new family samples but pointed to other regions, such as 7q36, 8p12, and 10q26, which warrant further investigation (Mustanski et al., 2005). These findings illustrate how polymorphic genes can contribute to genetic diversity and influence traits related to masculinity and femininity, potentially leading to variations in sexual preferences or behaviors.

Despite the progress made, further studies focusing on genetic markers and linkage analysis are needed to better identify the genetic factors associated with sexual behavior, which would deepen our understanding of its development and variability among individuals. The Xq28 region, for instance, includes several genes that may be relevant to sexual orientation. For example, the arginine vasopressin receptor (AVPR) 2 has been linked to social and affiliative behavior (Ebstein et al., 2012), and the cyclic nucleotide-gated channel alpha 2 (CNGA2) plays a critical role in regulating odor-evoked socio-sexual behaviors in mice (Mandiyan et al., 2005).

#### 3.2.2 The COMT and MTHFR genetic variants

Studies have focused extensively on a very important neurotransmitter in sexual behavior which is dopamine (Liu et al., 2008; Hull et al., 2004). Dopamine (DA), also referred to as 3,4-dihydroxytyramine, is a neurotransmitter produced by dopaminergic neurons in the brain. It is synthesized through the action of the enzyme tyrosine hydroxylase, which converts tyrosine into L-DOPA by adding a hydroxyl group. L-DOPA is then decarboxylated to form DA. The vesicular monoamine transporter 2 (VMAT2) transports DA into synaptic vesicles, from which it is released into the synaptic cleft and binds to dopamine receptors (DARs). DA interacts with five receptor subtypes—D1 to D5—all of which are part of the G protein-coupled receptor (GPCR) family (Speranza et al., 2021).

Whether DA exerts an excitatory or inhibitory effect depends on the type of receptor present on the target neuron’s membrane and the cell’s response to changes in cyclic AMP (cAMP) levels. Consequently, the various roles of DA are determined by the receptor subtype, cell type, synaptic characteristics, and interactions with other neurotransmitters (Speranza et al., 2021).

Groundbreaking research in the 1970s first identified dopamine’s role in penile erection and sexual behavior (Melis et al., 2022). This was further confirmed in 1986 when it was discovered that dopamine agonists, known for inducing penile erection by activating dopamine D2 receptors in male rats, produced the same effect when microinjected into the paraventricular nucleus (PVN) of the hypothalamus (Melis et al., 1987). Additionally, dopamine was shown to enhance copulatory behavior when injected into the medial preoptic area (Hull et al., 1986). This provided the first evidence that activation of the incertohypothalamic dopaminergic system, whose neurons originate in the A13 and A14 catecholaminergic cell groups of the hypothalamus (Dahlstroem and Fuxe, 1964), plays a key role in facilitating both penile erection and sexual behavior. More recent findings indicate that other dopaminergic systems, such as the mesolimbic and mesocortical pathways, are also involved in sexual behavior. These systems, with neurons located in the ventral tegmental area and projecting to the nucleus accumbens and medial prefrontal cortex, are critical for motivational and reward processes, including those related to sexual activity (Melis et al., 2022).

When dopaminergic (DAergic) neurons are activated, synaptic vesicles release their contents into the synaptic cleft, allowing dopamine (DA) to interact with postsynaptic DA receptors or presynaptic DA autoreceptors (Werkman et al., 2006; Zhang and Sulzer, 2012). To terminate the signal, extracellular DA must be cleared from the synaptic cleft. This can occur either through reuptake by DAergic neurons for recycling or through uptake by glial cells for degradation (Meiser et al., 2013). In glial cells, DA is rapidly broken down by monoamine oxidase (MAO) and catechol-O-methyltransferase (COMT). COMT gene is located on chromosome 22, specifically at 22q11.21 (Männistö and Kaakkola, 1999). COMT transfers methyl groups from S-adenosylmethionine (SAM) to the hydroxyl groups of catecholic compounds (Männistö et al., 1992; Männistö and Kaakkola, 1999). The 3-O-methylation of DOPAC by COMT produces homovanillic acid (HVA), a primary degradation product of DA. While COMT activity is present in glial cells, it is absent in the dopaminergic nigrostriatal neurons (Myöhänen et al., 2010).

A positive relationship between dopamine levels and male–male courtship has been established in animals’ studies (Cibrian-Llanderal et al., 2012; Chen et al., 2012; Liu et al., 2009; Liu et al., 2008). Several studies support the notion that genes influencing the dopaminergic pathway, like the COMT gene involved in dopamine metabolism and the methylenetetrahydrofolate reductase (MTHFR) gene which might influence COMT function through DNA methylation, make male more susceptible to be homosexual (Männistö and Kaakkola, 1999; de Arruda et al., 2013; Friso et al., 2002).

The COMT enzyme breaks down dopamine and norepinephrine (Männistö and Kaakkola, 1999; Sesack et al., 1998). Polymorphisms in the COMT gene have a direct impact on enzyme activity and estrogen levels leading to changes in biological traits linked to dopamine and estrogen (Camara et al., 2010; Calati et al., 2011). MTHFR is an important enzyme in carbon and folate metabolism and the synthesis of DNA and proteins (Nazki et al., 2014). It is mapped on chromosome 1 at the end of the short arm (1p36.6) (Liew and Gupta, 2015). One study explored specific genetic variabilities in COMT (rs4680, rs4818, and rs6267) and MTHFR (rs1801133) associated with male sexual orientation. Certain specific genetic variations like (MTHFR rs1801133 and COMT rs4818) were found to correlate with male homosexuality especially in homozygous comparisons (T/T vs. C/C at rs1801133; G/G vs. C/C at rs4818,) (Qin et al., 2018). However, in heterozygote comparisons (G/A vs. G/G), the variation in COMT gene at rs4680 indicated a tendency towards reducing the susceptibility to male homosexuality. This underlines the importance of the influence of COMT and MTHFR in male homosexuality susceptibility (Ramagopalan et al., 2010; Mustanski et al., 2005; Melis and Argiolas, 1995; Hull et al., 2004; Cibrian-Llanderal et al., 2012; Chen et al., 2012; Liu et al., 2009).

Genetic polymorphisms in the MTHFR gene might affect COMT methylation and function (Wang et al., 2015). The interaction between the COMT and MTHFR genes may influence male homosexuality through dopamine regulation. However, according to the study by Wang et al. (2015) there was no significant interaction between the two genes affecting this trait.

The interaction between childhood abuse experiences and specific polymorphisms in the COMT and MTHFR genes can influence the development of male homosexuality.

Stress leads to the triggering of excitement by the body’s sympathetic nervous system and raises the secretion of hormones from the pituitary and adrenal glands, such as cortisol (Szyf and Bick, 2013). COMT and MTHFR genes regulate the hypothalamic–pituitary–adrenal axis (Männistö and Kaakkola, 1999; de Arruda et al., 2013; Friso et al., 2002; Sesack et al., 1998). Therefore, by affecting the body’s stress response system, it is suggested that childhood abuse experiences and variations in the COMT and MTHFR genes influence the development of male homosexuality (Andersen et al., 2014; Edwards et al., 2003).

#### 3.2.3 The role of fragile X mental retardation 1 neighbor

Ladd et al. (2007) investigated the role of the fragile X mental retardation 1 neighbor (FMR1NB) in neuronal development and found that it interacts with proteins involved in synaptic function, suggesting its potential role in synaptic plasticity. Using bioinformatics analysis, a study by Hu et al. (2021) explored the potential association of the gene FMR1NB, with male sexual orientation via CRISPR mediated knockout mice. Rs17320865 is the primary single nucleotide polymorphism (SNP) found within the FMR1NB gene. This gene is known to affect brain function and development and has been used to understand dyslexia (Huc-Chabrolle et al., 2013; Liu et al., 2011). FMR1NB was linked previously to sexual preference. However, in this study, knock out mice in which the FMR1NB gene was absent displayed a tendency towards same-sex mounting, making a certain correlation with male sexual orientation (Hu et al., 2021).

#### 3.2.4 The role of ZNF536 gene

Rs7259428 is a single nucleotide polymorphism (SNP) located in the intron of the ZNF536 gene, which belongs to the zinc finger protein family—a group of transcription factors. ZNF536 is predominantly expressed in the brain, particularly in the dorsal root ganglia, cerebral cortex, hippocampus, and hypothalamus, including the suprachiasmatic nucleus (SCN) (Qin et al., 2009). This gene plays a crucial role in various cellular processes, such as gene regulation, transcriptional control, and chromatin remodeling. Notably, studies have shown lower expression of ZNF536 in the SCN region of homosexual individuals compared to heterosexual individuals.

Another study revealed significant genome-wide linkage, particularly on chromosomes 5q31 and 8q24, through the initial Genome-Wide Linkage Study (GWLS) on Cognitive Genes Network (CGN) in males. However, there was no overlap between the strongest linkage peaks for CGN and the most powerful signals identified in previous GWLS studies on male sexual orientation (Sanders et al., 2015). Although CGN and sexual orientation are related, they exhibit distinct and complex genetic patterns.

In a separate study, two loci (5q31 and 10q23) reached genome-wide significance (*p* < 5 × 10^–8^) in relation to CGN. Several other regions also showed positive associations (*p*-values ranging from 10^–6^ to 10^–8^). The SNP cluster on 5p13 includes the Solute Carrier Family 1 Member 3 (SLC1A3) gene, while the SNP cluster on 10q23 is associated with the Glutamate Receptor Delta 1 (GRID1) gene. SLC1A3, a glutamate transporter primarily expressed in the brain, is linked to various behavioral traits, including Attention Deficit Hyperactivity Disorder (ADHD), mood disorders, and corticolimbic connectivity during affective regulation (Huang et al., 2019; Medina et al., 2016; Poletti et al., 2018; van Amen-Hellebrekers et al., 2016). Additionally, disruptions in GRID1 have been associated with emotional and social behavioral alterations (Yadav et al., 2012). The complex relationship between GRID1, SLC1A3, and homosexuality suggests that while these genes are not directly responsible for sexual orientation, they contribute to the broader genetic and neurobiological framework influencing cognitive and behavioral traits, which may include sexual orientation. Their impact on brain function and emotional regulation plays a role in shaping complex behaviors and preferences.

#### 3.2.5 Association of homosexuality with chromosomes 7 and 8

In the first genome-wide screening study on male sexual orientation, Mustanski et al. (2005) analyzed 403 microsatellite markers spaced at 10 cM intervals across 146 families with two or more homosexual brothers. They identified a significant association between chromosome 7q36 and homosexuality, though this finding has yet to be confirmed by follow-up studies (Mustanski et al., 2005). Their findings also indicated a possible linkage to the pericentromeric region of chromosome 8 (approximately 60–90 cM, ~8p21–p11) (Mustanski et al., 2005). This locus was further supported by a genome-wide analysis of 409 independent pairs of homosexual brothers (Sanders et al., 2015) and a recent GWAS involving 1,077 homosexual men and 1,231 heterosexual men (Sanders et al., 2017). This region contains several candidate genes involved in the regulation of gonadal hormone levels. For instance, the gene GNRH1, located at 8p21, is secreted by hypothalamic neurons and stimulates the synthesis and release of LH and follicle-stimulating hormone (FSH). This gene plays a crucial role in sex-specific sexual development and behaviors (Desaulniers et al., 2017). Additionally, a gene located at 8p11.23 encodes the steroidogenic acute regulatory protein (STAR), which facilitates pregnenolone synthesis and is involved in the hypothalamic–pituitary regulation of adrenal steroid production (Sugawara et al., 1995). STAR plays a critical role in the development of congenital lipoid adrenal hyperplasia, a potentially life-threatening form of CAH (Fujieda et al., 1997; Nakae et al., 1997; Sahakitrungruang et al., 2010).

#### 3.2.6 Association of homosexuality with chromosomes 13 and 14

The utilization of Genome-Wide Association Screening (GWAS) allowed the identification of multiple single nucleotide polymorphisms (SNPs) on chromosomes 13 and 14 with potential links to male homosexuality. More recently, research analyzing large-scale datasets comprising 477,522 individuals identified five loci significantly associated with same-sex behavior, suggesting that genetic factors account for 8 to 25% of the variation in same-sex sexual behavior (Sanders et al., 2017; Ganna et al., 2019).

One region on chromosome 13, located between the genes SLIT and NTRK-like family member 6 (SLITRK6) and SLITRK5, has been proposed to be associated with male homosexual orientation (Sanders et al., 2017). SLITRK6 and SLITRK5 are primarily expressed in neural tissues (Aruga, 2003). In mice, SLITRK6 knockout leads to deafness and myopia, while in humans, SLITRK6 mutations result in severe myopia and sensorineural deafness (Tekin et al., 2013). Additionally, SLITRK5 deficiency impairs corticostriatal neurotransmission, leading to obsessive-compulsive-like behaviors in mice (Shmelkov et al., 2010). However, further research is needed to determine whether deficiencies in Slitrk5 or Slitrk6 influence sexual behaviors in mice or humans.

Several SNPs on chromosome 14 have also been associated with male homosexual orientation (Ramagopalan et al., 2010; Sanders et al., 2017). Specifically, genetic variation in intron 1 of the thyroid-stimulating hormone receptor (TSHR) gene on chromosome 14 has been suggested to explain the link between familial atypical thyroid function and male homosexuality. TSHR is critical for thyroid cell metabolism (Kleinau et al., 2013). Interestingly, an increased incidence of Graves’ disease, characterized primarily by hyperthyroidism, has been observed in homosexual men (Frisch et al., 2014). Additionally, a retrospective chart review indicated that gestational thyroid dysfunction may be associated with homosexual attraction in men (Sabuncuoglu, 2015). TSHR is expressed in both the thyroid gland and neuron-rich areas of the brain, such as the hippocampus (Crisanti et al., 2001), suggesting its potential role in regulating complex behaviors.

Overall, family and twin studies, along with genetic linkage and association analyses, provide evidence for candidate genetic variants on several chromosomes that may contribute to the development of male sexual orientation. These findings offer valuable clues for subsequent molecular genetic studies, although specific genes controlling sexual orientation have yet to be identified (see Tables 1, 2).

**Table 1:** Genes and potential association with homosexuality.

Gene	Role	Allele	Association with homosexuality and possible mechanisms	References
COMT	COMT: metabolism of catecholamine neurotransmitters such as dopamine, epinephrine, and norepinephrine	COMT rs4818, COMT rs4680, COMT rs6267	Genetic polymorphisms in the MTHFR gene could affect COMT methylation and function. The interaction between these genes may contribute to dopamine dysregulation, which is implicated in the mechanism underlying male homosexuality. COMT enzyme breaks down dopamine, and polymorphisms in the COMT gene can affect enzyme activity and estrogen levels. Changes in dopamine levels and estrogen can influence biological traits related to sexual orientation. Childhood abuse experiences can trigger stress responses in the body, involving the hypothalamic–pituitary–adrenal axis. COMT and MTHFR genes regulate this stress response system. Variations in these genes, combined with childhood abuse experiences, may influence the development of male homosexuality through their effects on stress response mechanisms.	Andersen et al. (2014), Calati et al. (2011), Camara et al. (2010), Chen et al. (2012), Cibrian-Llanderal et al. (2012), de Arruda et al. (2013), Edwards et al. (2003), Friso et al. (2002), Hull et al. (2004), Liu et al. (2009), Männistö and Kaakkola (1999), Männistö et al. (1992), Männistö and Kaakkola (1999), Melis and Argiolas (1995), Mustanski et al. (2005), Myöhänen et al. (2010), Qin et al. (2018), Ramagopalan et al. (2010), Sesack et al. (1998), Szyf and Bick (2013), Wang et al. (2015)
MTHFR	MTHFR: carbon and folate metabolism and the synthesis of DNA and proteins	MTHFR rs1801133		Andersen et al. (2014), Chen et al. (2012), Cibrian-Llanderal et al. (2012), de Arruda et al. (2013), Edwards et al. (2003), Friso et al. (2002), Hull et al. (2004), Liu et al. (2009), Männistö and Kaakkola (1999), Melis and Argiolas (1995), Mustanski et al. (2005), Nazki et al. (2014), Qin et al. (2018), Ramagopalan et al. (2010), Sesack et al. (1998), Wang et al. (2015)
FMR1NB	rs17320865	Brain function and development Synaptic plasticity	Dysregulation in synaptic plasticity can impact various neurological functions, including those related to sexual orientation. CRISPR knockout mice show correlation with male sexual orientation	Hu et al. (2021), Huc-Chabrolle et al. (2013), Ladd et al. (2007), Liu et al. (2011)
ZNF536	rs7259428	Gene regulation Transcriptional control Chromatin remodeling	Transcription factor with lower expression in homosexual individuals in the suprachiasmatic nucleus	Qin et al. (2009)

**Table 2:** Chromosomes and potential association with homosexuality.

Chromosome	Locus	Association with homosexuality	References
X chromosome	Xq28	Implication in sexual antagonism	Camperio-Ciani et al. (2004), Gavrilets and Rice (2006), Hamer et al. (1993), Mustanski et al. (2005)
Chromosomes 7, 8 and 10	7q36, 8p12, 10q26	Implication in sexual antagonism	Desaulniers et al. (2017), Fujieda et al. (1997), Mustanski et al. (2005), Nakae et al. (1997), Sahakitrungruang et al. (2010), Sanders et al. (2015), Sanders et al. (2017), Sugawara et al. (1995), C(5)

However, it is worth noting that the link between genetics and homosexuality is a complex field. Like all research related to homosexuality in humans, the main limitation is defining the trait. Some studies use a behavioral approach while others rely on the subjects’ definition of sexuality. Therefore, that definition not only differs between two different studies, but also among the subjects of the same research.

There is no single “gay gene,” there are multiple genes of interest involved making genetic testing in itself another limitation as studies are difficult to replicate. For example, genome-wide association studies vary greatly between populations and genes with small effects cannot be detected. Pleiotropy, the phenomenon where one gene locus can affect different phenotypic traits, can also be confounding in genetic research. One gene affecting multiple traits makes causality difficult to determine and lowers the ability to replicate research. The factors mentioned show why genetic testing in homosexuality is such an intricate field. Research fails in replicating previous results (as mentioned in Section 3.2.4 when discussing CGN and sexual orientation) and some studies are yet to be confirmed or reinforced (as mentioned in Section 3.2.5 when discussing chromosome 7q36).

### 3.3 Neurologic factors

Like various other behaviors, one can understand sexual orientation as a complex interaction involving specific brain processes. These processes involve at least three levels: (1) perception, which involves the feeling of attraction triggered by sensory perception, (2) self-identity, where an individual recognizes this attraction as part of their own identity, and (3) conscious actions directed towards individuals of their desired sex.

The potential biological underpinnings of male homosexuality are likely concentrated in midline regions of the brain. This is especially interesting because these specific brain areas, which include the hypothalamus and thalamus, are known for their involvement in processing sexual arousal (Poeppl et al., 2016; Kim et al., 2006; Motofei and Rowland, 2014). The midline regions of the brain also include the precuneus; it acts as a central hub with robust connections to various cortical regions, including the cuneus and the occipito-temporo parietal junction, which are involved in processing bodily cues. Moreover, the precuneus is linked to the ACC, a region responsible for self-perception also in decision making (Northoff, 2011), as well as subcortical structures that regulate sexual arousal (Poeppl et al., 2016; Kim et al., 2006; Motofei and Rowland, 2014; Cavanna and Trimble, 2006). Consequently, the precuneus may play a pivotal role in shaping choices related to sexual behavior. The findings do not support the idea that the male brain of homosexual individuals is uniformly feminized since there were distinctions in cortical thickness (Cth) and functional connectivity within the precuneus between male and female control groups. These distinctions may hold relevance for understanding sexual behavior.

A 2018 study by Manzouri and Savic employed MRI scans to investigate potential distinctions in brain structure and function among homosexual men (HoM), heterosexual men (HeM), and heterosexual women (HeW). The study was centered on regions of the brain previously identified as having gender-related variations. The primary objective was to determine whether there were any atypical brain distinctions in HoM, and if so, whether these variances were widespread, potentially contributing to behaviors more commonly associated with females in HoM or concentrated in networks related to self-referential sexual arousal.

The analysis encompassed measures such as cortical thickness (Cth), surface area (SA), volumes of subcortical structures, and the functional connectivity observed during resting states in 30 HoM, 35 HeM, and 38 HeW. Findings revealed that HoM exhibited significantly greater thickness in specific brain regions, including the anterior cingulate cortex (ACC), precuneus, and the left occipito-temporal cortex when compared to both control groups. Notably, these differences seemed to be interrelated, as HoM also showed more pronounced associations between these cortical areas.

Furthermore, within the default mode network, responsible for self-referential cognitive processes and inclusive of the ACC and precuneus, HoM exhibited weaker functional connections compared to HeM and HeW. Conversely, functional connectivity between the thalamus and hypothalamus, which are crucial for sexual behavior, was stronger. In addition to these distinctive features, HoM displayed brain characteristics that resembled those typically associated with “female” brain structures, such as similar cortical thickness in the left superior parietal and cuneus cortices to HeW, distinguishing them from HeM (Manzouri and Savic, 2018).

Cth serves as an indicator of neuronal attributes such as size, number, shape, density, and dendritic connections within the columns of the cortex (Rakic, 1988). It can be utilized as a proxy measure for assessing the overall condition of the cerebral cortex. On one hand, it has been observed that the parietal and superior frontal lobe cortex in male controls is thinner compared to that of female controls. This thinning demonstrates a reverse relationship with testosterone levels (Savic and Arver, 2014; Nguyen et al., 2013; Bramen et al., 2012). On the other hand, several studies have shown that the left superior temporal and occipital cortices in male controls are thicker than those in female controls, and this increased thickness is positively associated with testosterone levels (Herting et al., 2015).

Hence, it is reasonable to consider that the thicker parietal and frontal lobe cortex, along with the thinner cuneus cortex observed in homosexual men, may suggest lower androgen levels in specific brain regions. By contrast, homosexual participants demonstrated normal testosterone levels which means that this potential reduction in androgenization could imply a diminished expression or function of androgen receptors (Raznahan et al., 2010).

Neurodevelopmental processes are likely to wire neural circuits differently in individuals with same-sex attractions compared to those with opposite-sex attractions. The initial evidence pointing to neural correlates of sexual partner preference comes from Simon LeVay’s 1991 autopsy study of the third interstitial nucleus of the anterior hypothalamus (INAH-3). LeVay observed that homosexual men had a smaller INAH-3 size in comparison to presumed heterosexual men, resembling the size found in presumed heterosexual women. It’s important to note that the structural distinctions within same-sex groups may not be particularly pronounced.

#### 3.3.1 The role of hypothalamus

A positron emission tomography (PET) study has indicated a more robust hypothalamic response to serotonin in heterosexual men compared to homosexual men (Kinnunen et al., 2004). Furthermore, neuroimaging investigations comparing heterosexual men and women while exposed to their preferred sexual imagery have demonstrated significantly greater hypothalamic activation in heterosexual men (Karama et al., 2002). Taken together with the earlier observations of a smaller INAH-3 in homosexual men, these findings suggest the existence of a distinct functional foundation in the anterior hypothalamus for sexual attraction toward women. Additional support for this concept arises from studies involving lesion models of the preoptic area (POA) within the anterior hypothalamus, which have revealed reduced responses of male animals toward females (Hull et al., 2002).

While discussing the hypothalamus, aromatase and androgens must be mentioned as well since animal models indicate that prenatal androgens may have a role in shaping sexual variations in hypothalamic regions (Morris et al., 2004). For instance, in specific sheep species, some males exclusively prefer the same sex. These males also exhibit reduced aromatase activity and smaller ovine sexually dimorphic nuclei (homolog to the human INAH-3) compared to sheep oriented towards females (Roselli et al., 2004).

Moreover, potential disparities in aromatase activity in human males based on their sexual orientation may help elucidate the complex mix of masculine and feminine traits often observed. For instance, a decline in aromatase activity in homosexual men, as implied by the Roselli study, could lead to reduced availability of aromatized testosterone, particularly estradiol, which is known to contribute to the masculinization of the male mammalian brain (Morris et al., 2004). This could result in less masculinized hypothalamic circuitry, while simultaneously allowing excess non-aromatized testosterone to contribute to the masculinization of other androgen-sensitive traits, such as the 2D:4D ratio, through alternative metabolic pathways (e.g., 5-alpha reductase). Research on hypothalamic structure and function, particularly LeVay’s early work, is constrained by small sample sizes, the use of post-mortem brains, and assumptions regarding the sexual orientation of subjects. Moreover, other confounding factors—such as comorbid medical conditions or the influence of hormone treatment—may contribute to the observed anatomical differences.

#### 3.3.2 The connection between sexual orientation and cognitive processes

Research in neurocognition has provided evidence indicating that differences in brain activity related to sexual orientation might also manifest in higher cortical regions. Multiple independent studies demonstrate that homosexual men tend to perform poorly on fundamental tests of spatial abilities, such as mental rotation and spatial perception, compared to heterosexual men (Rahman and Wilson, 2003a, 2003b). Moreover, homosexual men display heightened spatial location memory, improved recall of spatial landmarks during navigation, and enhanced phonological and semantic fluency—traits typically associated with females—compared to heterosexual men (Rahman et al., 2003a, 2003b, 2003c). These findings suggest that sexual orientation could be linked to variations in brain regions like the parietal, hippocampal-temporal, and prefrontal areas that underlie the mentioned cognitive abilities. Variations in inter-hemispheric pathways might contribute to these cognitive differences but they could not be well replicated (Allen and Gorski, 1992; Wegesin, 1998a, 1998b). A study found a difference in the size of the callosal isthmus between right-handed homosexual and heterosexual men, with homosexual men exhibiting a larger isthmus (Witelson et al., 2008). This finding suggests that right-handed homosexual men may possess brain structures and organization typically seen in left-handers, despite their apparent right-hand preference (Witelson et al., 2008). This anatomical evidence supports the notion that these men might exhibit atypical functional asymmetry. These results align with previous neuropsychological studies, which showed that right-handed homosexual men demonstrate less functional asymmetry compared to their heterosexual counterparts in verbal dichotic tasks (McCormick and Witelson, 1994), divided visual-field tasks (Sanders and Wright, 1997), and neurophysiological EEG measures (Wegesin, 1998a, 1998b).

Further investigations employing various neurophysiological measures provide additional evidence supporting the involvement of parietal and temporal lobes (Howard et al., 1994; Reite et al., 1995; Wegesin, 1998a, 1998b). The parietal lobe seems to be implicated given its role in neural processes related to heterosexual sexual arousal and the visual-configural processing of preferred sexual stimuli (Howard et al., 1994; Waisman et al., 2003). The pre-pulse inhibition of the startle response (a neurobehavioral probe of sexual dimorphism) is notably more masculinized in homosexual women compared to heterosexual women, pointing to the involvement of the pallido-striato-thalamic limbic circuitry (Rahman et al., 2003a, 2003b, 2003c). Cognitive studies indicate that homosexual women tend to exhibit better verbal fluency, suggesting the involvement of the prefrontal cortex, although neurophysiological studies do not consistently reveal differences, except in auditory regions (McFadden and Champlin, 2000). Cognitive and neuroanatomical differences observed between homosexual and heterosexual individuals are intriguing but not yet conclusive. Many studies suffer from small sample sizes and limited replication. Additionally, observed brain differences may be shaped by social experience and plasticity rather than reflecting innate or causal factors (see Table 3).

**Table 3:** Summarizing the neurological factors and brain regions implicated in various aspects of homosexuality, as discussed in the provided text.

Neurological level/factor	Key brain regions involved	Description of role/findings	References
Sexual orientation processing	Hypothalamus, thalamus, precuneus, anterior cingulate cortex (ACC), cuneus, occipito-temporo parietal junction	Sexual orientation involves sensory perception of attraction, self-identity, and conscious actions. Midline brain regions, including the hypothalamus and thalamus, are crucial for sexual arousal. The precuneus acts as a central hub connecting to regions for bodily cue processing and is linked to self-perception (ACC) and sexual arousal regulation.	Poeppl et al. (2016), Kim et al. (2006), Motofei and Rowland (2014), Northoff (2011), Cavanna and Trimble (2006)
Brain structure (cortical thickness and connectivity)	Anterior cingulate cortex (ACC), precuneus, left occipito-temporal cortex, left superior parietal cortex, cuneus, default mode network, thalamus, hypothalamus	Homosexual men (HoM) show greater thickness in ACC, precuneus, and left occipito-temporal cortex compared to heterosexual men (HeM) and heterosexual women (HeW), with more pronounced associations between these areas. HoM exhibit weaker functional connections within the Default Mode Network but stronger connections between the thalamus and hypothalamus. Some HoM brain characteristics resemble “female” structures (e.g., similar cortical thickness in left superior parietal and cuneus cortices to HeW). Cortical thickness (Cth) reflects neuronal attributes.	Manzouri and Savic (2018), Rakic (1988), Savic and Arver (2014), Nguyen et al. (2013), Bramen et al. (2012), Herting et al. (2015)
Androgen levels and receptors	Parietal and superior frontal lobe cortex, cuneus cortex	Thicker parietal and frontal lobe cortex and thinner cuneus cortex in homosexual men may suggest lower androgen levels in specific brain regions. While overall testosterone levels may be normal, this could imply diminished expression or function of androgen receptors.	Savic and Arver (2014), Nguyen et al. (2013), Bramen et al. (2012), Herting et al. (2015), Raznahan et al. (2010)
Neurodevelopmental wiring (INAH-3)	Third interstitial nucleus of the anterior hypothalamus (INAH-3)	Initial evidence suggests different neural circuit wiring. LeVay’s 1991 autopsy study found that homosexual men had a smaller INAH-3, comparable to heterosexual women, but structural distinctions within same-sex groups may not always be pronounced.	Levay (1991)
Hypothalamic function and hormonal influences	Hypothalamus (especially anterior hypothalamus, preoptic area—POA)	Heterosexual men show a more robust hypothalamic response to serotonin and greater hypothalamic activation to preferred sexual imagery compared to homosexual men. Lesion studies of the POA in animals reduce male attraction to females. Prenatal androgens and aromatase activity (which converts testosterone to estradiol) may shape sexual variations in hypothalamic regions; e.g., reduced aromatase activity in some homosexual sheep models is linked to smaller sexually dimorphic nuclei and same-sex preference.	Kinnunen et al. (2004), Karama et al. (2002), Hull et al. (2002), Morris et al. (2004), Roselli et al. (2004)
Cognitive processes and brain asymmetry	Parietal, hippocampal-temporal, prefrontal areas, corpus callosum (callosal isthmus), pallido-striato-thalamic limbic circuitry, auditory regions	Homosexual men often perform poorer on spatial ability tests but show heightened spatial location memory and improved verbal fluency (traits often associated with females) compared to heterosexual men. These suggest variations in underlying brain regions. Homosexual men, especially right-handed ones, may exhibit atypical functional asymmetry, with some studies showing a larger callosal isthmus. Homosexual women may show a more masculinized pre-pulse inhibition of startle response (implicating limbic circuitry) and better verbal fluency (suggesting prefrontal cortex involvement).	Rahman and Wilson (2003a, 2003b), Rahman et al. (2003a, 2003b, 2003c), Allen and Gorski (1992), Wegesin (1998a, 1998b), Witelson et al. (2008), McCormick and Witelson (1994), Sanders and Wright (1997), Howard et al. (1994), Reite et al. (1995), Waisman et al. (2003), McFadden and Champlin (2000)

### 3.4 Environmental factors

#### 3.4.1 Childhood gender nonconformity (CGN)

##### 3.4.1.1 Play preferences and gender-typed behaviors

Scientific research has demonstrated a strong link between sexual orientation and various aspects of gender roles. This encompasses childhood behaviors such as play preferences and the formation of gender identity (Bailey and Zucker, 1995), as well as gender-typed behaviors in adulthood, particularly in terms of career choices and leisure activities (Lippa, 2008).

Recent large-scale studies involving population-based prospective research have confirmed the robust association between sexual orientation and Childhood Gender Nonconformity (CGN) (Li et al., 2017; Xu et al., 2019, 2020, 2021). Another study involved assessing behaviors observed in home videos from childhood. This study has found that, consistent with the typical development of gender identity, variations in CGN as assessed by observers become apparent around the age of 3 or 4 (Rieger et al., 2008a, 2008b).

##### 3.4.1.2 Childhood gender nonconformity in males

An alternate method has explored the relationship between homosexual men’s recollections of CGN and their mothers’ memories of their sons and found a strong correlation between the two (r = 0.69) (Bailey et al., 1995). This suggests that retrospective accounts from both individuals and their parents are reliable indicators of past behaviors, supporting the idea that CGN is a noticeable and significant aspect of childhood that may be linked to the later development of sexual orientation. The study also underscored the potential impact of parental perception and societal expectations on how gender-nonconforming behavior is recognized and remembered, contributing to the broader understanding of the factors that influence sexual identity development. The connection between sexual orientation and CGN remains consistent across different cultures (Bailey et al., 2016). A meta-analysis of 41 retrospective studies revealed that individuals with a homosexual orientation recalled significantly more instances of CGN compared to their heterosexual counterparts (Bailey and Zucker, 1995).

In a study involving families, there were moderate family patterns and consistent differences in CGN related to sexual orientation among males (Bailey et al., 2020). It’s noteworthy that twin studies indicate that CGN appears to have a moderate to high heritability among males, with heritability estimates ranging from 29 to 70% (Knafo et al., 2005; van Beijsterveldt et al., 2006; Alanko et al., 2010; Bailey et al., 2000).

Much of the evidence linking childhood gender nonconformity to adult sexual orientation relies on retrospective self- or parent-report, which may be subject to memory and social desirability biases. Furthermore, cultural expectations regarding gender roles may shape both the perception and expression of such behaviors, complicating their interpretation across different contexts.

#### 3.4.2 Early exposure to traumatic events

The development of homosexuality can be influenced by early exposure to traumatic events. These include neglect, sexual, emotional, or physical abuse (Qin et al., 2018). However, the mechanism relating those two variables remains unclear and requires further investigation. Certain studies have shown that some stressful events that occur in life influence both mental and physical health and can impact the development of homosexuality. Male homosexuals declare more incidence of childhood abuse compared to heterosexuals as reported by studies conducted by Wyre (1990), Rind (2001), and Garcia et al. (2002). The abuse episodes have played a role in shaping sexual orientation. Therefore, childhood victimisation is a very sensitive concern since it can have long lasting negative effects on mental and physical health. Childhood victimisation events occur early during a critical period in a child’s brain development and at that time the stress response is extremely sensitive to variables such as abuse, neglect, peer rejection and unstable family environment. It is speculated that all the factors listed previously in combination with the genetic factors like COMT and MTHFR genes collectively contribute to the development of male homosexuality (Qin et al., 2018). While some studies suggest an association between early trauma and later sexual orientation, causality cannot be established. The possibility of recall bias and the stigmatizing nature of such associations call for cautious interpretation. This line of research remains contentious and must be approached with sensitivity and scientific rigor.

A meta-analysis found no statistically significant difference in the overall prevalence of childhood sexual abuse (CSA) among lesbian, gay, and bisexual individuals, with this pattern observed in both men and women. Specifically, CSA prevalence among male sexual minorities was reported at 22.2%, while for female sexual minorities it was 36.2% (Xu and Zheng, 2015). These figures contrast markedly with estimates from heterosexual populations: Pereda et al. (2009b) reported CSA prevalence rates of 7.9% for men and 19.7% for women in a general population sample, and Stoltenborgh et al. (2011) found similar rates of 7.6% for men and 18.0% for women. Taken together, these findings suggest that CSA is more commonly reported among sexual minorities than among heterosexual individuals. However, it is important to emphasize that these data demonstrate an association rather than a definitive causal relationship between CSA and sexual orientation.

Some researchers have proposed that CSA could influence the development of same-sex attraction (Roberts et al., 2013). For example, same-sex sexual abuse during childhood may lead some boys to question or believe they are homosexual (Gartner, 1999), while sexual abuse of girls by male perpetrators may foster aversion toward men and a greater sense of comfort in intimate relationships with women (Marvasti and Dripchak, 2004). These hypotheses, while suggestive, remain the subject of ongoing debate and require further empirical investigation.

Although the meta-analysis sought to estimate the global prevalence of childhood sexual abuse (CSA) among lesbian, gay, and bisexual (LGB) populations, the majority of included studies were conducted in the United States and Canada. Additionally, there was a notable absence of research from African countries, many of which maintain laws criminalizing same-sex behavior, making it challenging to study sexual minorities in these regions. Furthermore, the meta-analysis was restricted to English-language publications, which likely limited the comprehensiveness of the included studies. Hence, the analysis was unable to clarify the temporal sequence between sexual orientation and CSA, meaning that it remains unclear which factor precedes the other (see Table 4).

**Table 4:** Summarizing the environmental factors which have been linked to sexual orientation development.

Environmental factor	Key brain regions involved	Description of role/findings	References
childhood gender nonconformity (CGN)	Play preferences and gender-typed behaviors: preference for toys/activities typical of the opposite sex; adopting gender-typed behaviors in adulthood (e.g., career choices, leisure).	Strong, consistent association between sexual orientation and CGN across large-scale prospective studies and cultural contexts. Observable around age 3–4. Retrospective accounts from homosexual men and their mothers show strong correlation regarding CGN, suggesting reliability. Twin studies indicate moderate to high heritability (29–70%) for CGN in males, but environmental factors also play a role.	Bailey and Zucker (1995), Li et al. (2017), Xu et al. (2019, 2020, 2021), Rieger et al. (2008a, 2008b), Bailey et al. (1995, 2016, 2020), Knafo et al. (2005), van Beijsterveldt et al. (2006), Alanko et al. (2010), Bailey et al. (2000)
Early exposure to traumatic events	Neglect, sexual abuse, emotional abuse, physical abuse, peer rejection, unstable family environment.	Traumatic events in childhood are linked to the development of homosexuality, though the exact mechanism is unclear. Studies report higher incidence of childhood abuse among homosexual men compared to heterosexual men. These events occur during critical periods of brain development, potentially interacting with genetic factors (e.g., COMT, MTHFR genes) to influence sexual orientation. Childhood victimization has long-lasting negative effects on mental and physical health.	Qin et al. (2018), Wyre (1990), Rind (2001), Garcia et al. (2002)

## 4 Conclusion

In a narrower context, “sexual orientation” is concerned with an individual’s preference for a sexual partner. Homosexuality is a behavior exhibited among people of the same sex. This behavior is grounded in romantic as well as sexual attraction. The connection between homosexuality and polymorphic genes is supported by two distinct lines of evidence. First, twin studies point to the involvement of genetic factors contributing to homosexuality. Additionally, environmental factors play a role in shaping homosexual characteristics (as seen in Pillard and Bailey, 1998; Bailey et al., 1999; Dawood et al., 2000). Male homosexuality appears to exhibit a heightened inheritance pattern through the maternal lineage (as indicated in Pillard et al., 1981, 1982; Camperio-Ciani et al., 2004). Furthermore, the possibility of polymorphic X-linked genes influencing male homosexuality could also be present. The underlying causes of male and female homosexuality may differ. One basis for is the presence of a well-documented fraternal birth order effect in male homosexuality. In contrast, female homosexuality is independent of ‘sororal’ birth order (Bogaert, 1997).

Childhood abuse has been linked to homosexuality. Additionally, specific genetic variants might contribute to homosexuality. Thus, these factors could potentially have a positive correlation with homosexuality (Qin et al., 2018). These findings could provide valuable clues regarding the genetic foundations of male sexual orientation when considering a wider range of populations were linked to same-sex sexual behavior (Hu et al., 2021).

Understanding the complex interplay of biological, genetic, neurological, and environmental factors in shaping human sexual orientation remains a significant and evolving scientific challenge. Current research suggests that homosexuality cannot be attributed to a single cause but rather arises from a constellation of influences, including but not limited to genetic predispositions, hormonal environments in utero, brain structure differences, and psychosocial contexts. To advance knowledge in this field, interdisciplinary research is crucial. Future studies should integrate genomics, neuroimaging, endocrinology, and longitudinal psychosocial data while ensuring large, diverse, and representative samples. It is also important to study people from different backgrounds and use large, diverse groups in research. Scientists should work together across countries and fields and focus on making research more reliable and respectful of LGBTQ+ individuals. By doing this, we can improve knowledge, reduce prejudice, and help shape more inclusive policies and education.

This narrative review is subject to several inherent limitations. In contrast to systematic reviews, it does not adhere to a standardized, predefined protocol for identifying, selecting, and evaluating the literature, thereby increasing the risk of selection bias. The synthesis presented is shaped by the authors’ interpretations, and the absence of a formal quality appraisal of the included studies constrains the ability to determine the strength and reliability of the evidence. Research on homosexuality is marked by substantial heterogeneity, encompassing variations in study design, definitions of sexual orientation, and the tools used for measurement—factors that complicate both comparability and synthesis of findings. Cultural, political, and legal environments exert a significant influence on research practices and participants’ willingness to disclose personal information, potentially resulting in the underrepresentation of marginalized populations. Moreover, the evidence base is disproportionately drawn from Western, high-income settings, creating both geographical and cultural imbalances. Lastly, given the evolving societal understanding of sexual orientation and identity, some older studies may not align with contemporary conceptual frameworks, thereby limiting their present-day applicability.

## 5 Future perspective

To truly understand the origins of homosexuality, future research must delve deeper into specific biological, genetic, and neurological mechanisms, moving past broad correlations. This means conducting large-scale genome-wide association studies (GWAS) with diverse groups to pinpoint specific genetic links (Ngun and Vilain, 2014). We also need to explore epigenetic modifications, investigating how prenatal environmental factors might influence brain development and sexual orientation (Rice et al., 2012) Importantly, these studies should include sex-specific analyses, as pathways might differ for males and females (Ngun and Vilain, 2014).

In neurological research, advanced neuroimaging techniques, such as longitudinal fMRI and DTI studies, can examine brain structure and function in young individuals to identify early neural correlates of sexual orientation (American Psychological Association, 2008). Combining this brain data with genetic and hormonal information will offer a more comprehensive view of how these factors shape brain circuitry related to attraction.

For environmental influences, future studies should pinpoint specific prenatal and early postnatal exposures (e.g., in utero hormones, maternal stress) and their dose–response relationships (American Psychological Association, 2008). Understanding gene–environment interactions is also crucial, exploring how genetic predispositions might be expressed differently under various environmental conditions. This will require well-designed prospective cohort studies or controlled animal models.

Ultimately, a complete understanding requires integrative, multi-omics approaches combining genetic, epigenetic, proteomic, and neuroimaging data. It’s also vital to diversify study populations, refine phenotypic measures of sexual orientation, and use longitudinal study designs to track development over time, all while maintaining rigorous ethical standards (American Psychological Association, 2008; Ngun and Vilain, 2014; Rice et al., 2012). By pursuing these avenues, we can gain a more nuanced appreciation of homosexuality as a fundamental aspect of human diversity.
